# Completion rate of tuberculosis preventive therapy and incidence of tuberculosis among people living with the Human Immunodeficiency Virus on antiretroviral therapy in Ekurhuleni East subdistrict, Gauteng province

**DOI:** 10.11604/pamj.2024.48.86.43117

**Published:** 2024-07-04

**Authors:** Refiloe Mashego Malaka, Lindiwe Cele, Mabina Mogale, Thembi Simbeni

**Affiliations:** 1 Department of Public Health, Epidemiology and Biostatistics Unit, Sefako Makgatho Health Sciences University, Ga-Rankuwa 0208, South Africa,; 2 Health System Management and Policy Unit, Department of Public Health, Sefako Makgatho Health Sciences University, Ga-Rankuwa 0208, South Africa

**Keywords:** Tuberculosis preventive therapy initiation, tuberculosis preventive therapy completion, people living with HIV, antiretroviral therapy patients, tuberculosis preventive therapy

## Abstract

**Introduction:**

the World Health Organization (WHO) has recommended the use of tuberculosis preventive therapy (TPT) as part of a comprehensive care package for the reduction of tuberculosis (TB) incidence among people who are living with human immunodeficiency virus (PLWHA). When used optimally, TPT efficacy ranges between 60% and 90% among adults and children who are living with HIV. Despite the wide adoption of this intervention in South Africa, the country remains heavily burdened with high rates of TB/HIV co-infections, reported to be 59% in 2018. Reported challenges include low uptake and completion rates. This study aimed to determine the TPT completion rate and investigate the incidence of TB among antiretroviral therapy (ART) patients who were initiated on TPT.

**Methods:**

this descriptive cross-sectional retrospective cohort study was conducted among HIV-positive patients who were on ART, 18 years old and above, and had been initiated on TPT between June 2019 and June 2021 at the selected PHC facilities in Ekurhuleni East sub-District. We conducted record reviews and face-to-face interviews to collect data. These were captured onto a Microsoft Excel spreadsheet, cleaned, and coded before importation onto the Epiinfo version 7 statistical software package for statistical analyses.

**Results:**

the study found a majority of female participants, (60.5%). The median age of participants was 39.0 years (IQR=15), with most aged 50 years old and above, (21.3%). The treatment course of TPT was completed at the prescribed 12 months by 196 (30%) of the 395 participants. Only 12 (3%) of the participants were found to have TB, half 6 (50%) of which were breakthrough cases of TB. The reasons for non-completion of TPT included clinicians not offering it to patients, (46/276 (16.7%)). The barriers to TPT completion included not having a treatment supporter, (73.2%); p<0.001, while disclosure of positive HIV status was found to facilitate TPT completion (83.2%); p<0.001.

**Conclusion:**

the observed TPT completion rate of 30% needs to be addressed as it is far below the national threshold of 85%. The barriers and facilitators to TPT completion also require attention to help improve the TPT completion rate.

## Introduction

Tuberculosis (TB) is the leading cause of death among people who are infected with the human immunodeficiency virus (PLWHA). Individuals who are HIV positive have an increased risk of progression from latent TB to active TB disease. Hence, the World Health Organization (WHO) has recommended the use of TB preventive therapy (TPT) among PLWHA, including other groups of people who are considered at high risk of progression to active TB disease. These groups include children who are < 5 years old and are household contacts of people who have bacteriologically confirmed pulmonary TB (PTB), and other selected house contacts of people with multidrug-resistant TB (MDR-TB), [1,2]. Tuberculosis preventive therapy functions synergistically and independently of ART reducing TB-related mortality among PLWHA by 37%, thereby affording the PLWHA healthier and longer lives [3]. Despite its cost-effectiveness, the level of TPT use remains suboptimal, with global statistics showing a decline to 1.9 million PLWHA in 2022 from 2.2 million in 2021 and 2.4 million in 2020. These declines have been observed mostly among the countries that are in the WHO African region [4,5]. The rollout of TPT in South Africa occurred in the early 2000s, with the country´s national guideline restricting eligibility for TPT by prioritizing PLWHA and household contacts of people with TB who are 5 years old and younger [6,7].

South Africa has one of the highest TB and HIV burdens globally, with a TB/HIV co-infection rate of 59% in 2019, however implementation of this intervention is reported to be suboptimal, [8,4]. Tuberculosis preventive therapy completion rates were as low as 45.7% and 52,6% have been reported [9,10]. Several studies have reported an array of structural, staff, and patient-related barriers to TPT uptake and completion. These include infrastructure and inability to access healthcare facilities, as well as staff and treatment shortages [9,11]. Patient-related challenges that have been cited include treatment side effects, socio-economic factors, insufficient education about TPT to HIV-related stigma. Health care worker influences include inadequate understanding of TPT by the prescribing health care workers, doubt about the effectiveness of TPT, disregard of TPT as a priority in their practice, not sure of who is responsible for TPT implementation, ineffective contact tracing, lack of resources, and lack of standardized reporting tools have been reported [11,4]. The study aims to determine TB incidence and barriers to TPT completion among people living with HIV on ART. Several studies have been conducted on TPT uptake, however, there is a paucity of TPT completion studies, hence this study was conducted to determine the proportion of ART patients who develop TB among those who were initiated on TPT, the proportion of patients that complete 12 months of TPT course, and to investigate the barriers to TPT completion.

## Methods

**Study design:** this quantitative, descriptive, cross-sectional retrospective cohort study used health facility records to collect demographic and clinical data. Face-to-face interviews were conducted to collect sociodemographic data relating to barriers and facilitators of TPT completion among ART patients who were accessing HIV care at the selected PHC facilities in Ekurhuleni district.

**Study setting:** the study was conducted at public health care facilities located at Ekurhuleni East sub-District in the Gauteng Province of South Africa. Ekurhuleni is the economic hub of the Gauteng province and the largest population in the province and the country; with an estimated population of about 3,774,638 comprising 51% males. The area has a high unemployment rate of 31.6%, a figure that surpasses provincial and national unemployment rates. TB and HIV were among the top 10 leading causes in Gauteng in 2016 [12-14]. However, the latest health profiling of the subdistrict shows TB and HIV as the main cause of death among the females in the age group 15- and 24-year-olds die due to related diseases with the male counterparts dying mostly due to injuries [13]. Ekurhuleni has 30 primary healthcare facilities (PHCs) facilities and 2 district hospitals. These facilities operate from Monday to Friday between 07:30 and 16:30, with a few of them operating 24/7 and some doing extended hours on Saturday, from 07:00 to 14:00 [15].

**Data sources and target population:** the target population for this study was ART patients who were receiving ART care at the selected health facilities in the Ekurhuleni East sub-District. Participants were included if they were aged 18 years or older and had been on TPT for at least 12 months. They were excluded if they did not give consent for participation. Data collection was based on the DoH algorithm for the management and treatment of HIV-positive patients who are to initiate ART. This shows how patients who are to be initiated on ART are managed. This includes screening for TB before ART initiation, whereby patients are offered TPT if they do not have active TB, and how they are treated for TB if they are TB active. A researcher-developed questionnaire containing question items adapted from previous studies' data collection tools were used to collect socio-demographic information and data pertaining to barriers and facilitators to TPT completion [10,16,17]. The data collection tool was written in English and translated into Isizulu, the most spoken language in the area. The questionnaire was piloted on 10 participants who were conveniently sampled at one of the selected health facilities, and those participants were not included in the main study. No major amendments to the tool were required, except for a few questions the participants did not understand. Data collection occurred between April 2023 and August 2023, lasting approximately 18 weeks.

**Study sample size:** the sample size was determined using the Raosoft sample size calculator [18]. We considered the total headcount of 5,400 patients that were initiated on ART and TPT from each of the selected facilities between June 2019 and June 2021, as population size. We chose a 95% confidence level, which corresponds to a 5% margin of error, and selected a 50% response rate to obtain a sample size of 359. A 10% buffer was added to obtain a final sample size of 395. Health facilities were selected based on high headcount and accessibility and this included facilities in informal settlements and elite urban residential suburbs. Those that had a headcount of <1000 per month and those that were more than 35 km radius apart from each other were excluded. The contribution of each facility to the total headcount was determined, and the number of participants to sample from each health facility was calculated proportional to the required sample size.

**Participant selection and recruitment:** participants were selected using the simple random sampling (SRS) strategy from the numbered line lists of patients that were generated from the Tier. Net register. Recruitment occurred at the waiting area before consultation, using patient files that were pre-retrieved on the previous day. Identified participants were called to one consultation room where the study was introduced and those who were willing to participate were asked to sign the informed consent form (ICF). The interviews were conducted on the spot with those who were still awaiting the health service first, with others who were requested to come after consultation. Before conducting the interviews, the participants were given information about the study including the purpose of the study, and issues of anonymity and voluntariness. Each interview session lasted for about 25 to 30 minutes to complete.

### Variables

**Dependent variables:** this study has defined TPT completion based on whether the patient started the course of Isoniazid and took it for the prescribed period of 12 months. Tuberculosis incidence was defined based on whether the patient developed TB while they were on TB treatment or after TPT was completed. Independent variables: the independent variables included sex, age, marital status, highest level of education, residence, and employment. Marital status was defined as whether the participant was married or unmarried (divorced, living with a partner, separated, single, or widowed). The level of education was defined as whether the participant has no formal education or had (completed grade 12, Primary, secondary, or Tertiary). Residence was defined as the place where the participant was staying, whether in (the hostel, informal settlement, or township). Employment was defined by whether the participant was Unemployed or employed (full-time, part-time, self-employed). The data on independent variables were sourced from patient folders, Tier.net, and from the participants during the interview.

**Statistical analysis:** the Excel data file was imported onto the Epiinfo version 7 software package to conduct the statistical data analyses. We conducted univariate analysis of the sociodemographic, clinical, and barrier-related data. We used the mean and standard deviations (SD) or median and interquartile range (IQR) to present the numeric data. We further categorized the variable age of participants, and the age groups were presented as proportions and percentages, along with other categorical data such as gender, the level of education, and the reasons for non-completion of TPT. Frequency tables and figures were used to display the data. We conducted the bivariate analysis using question items relating to barriers and facilitators to TPT completion. These were presented as proportions and percentages with p values <0.05 used to indicate the statistical significance of the differences.

**Ethical approval:** the study obtained ethical clearance from the Research Ethics Committee of Sefako Makgatho Health Sciences University (SMUREC/H/392/2022: PG). Permission to conduct the study was granted by the Ekurhuleni Health District Research Committee (GP_202302_003). Written informed consent was obtained from the participants before participation. The data file was password-protected to ensure confidentiality.

## Results

**Sociodemographic characteristics of the study participants:** the results show that out of 395, 239 (60.5%) participants were females. The median age of participants was 39 years (IQR=15). The youngest and the oldest participants were respectively 19 and 99 years old, with 84 (21.3%) above the age of 50 years. Nearly half of the participants were single (191 (48.4%), and only 5 (1.27%) were divorced. Slightly more than half had completed grade 12 (222 (56.2%)), with only 4 (1%) who had no formal education. One hundred and eighty-two (46.1%) were unemployed with only 26 (6.6%)) who were self-employed. Most stayed in townships, (307 (77.7%)), and 13 (3.3%)) who stayed in hostels, (Table 1).

**Table 1:** sociodemographic characteristics of the study participants, N=395

Characteristic	Frequency	Percentage
**Sex**		
Male	239.0	60.5%
Female	156.0	39.5%
**Age**		
**Median (IQR)**	39.0 (15)	
**Age group**		
15-19	3.0	0.8%
20-24	15.0	3.8%
25-29	42.0	10.6%
30-34	66.0	16.7%
35-39	77.0	19.5%
40-44	65.0	16.5%
45-49	43.0	10.9%
50+	84.0	21.3%
**Number of dependents**		
Mean (SD)	2.1 (1.4)	
0-2	243.0	
3-5	146	
>5	6.0	
**Marital status**		
Divorced	5.0	1.3%
Living with partner	106.0	26.8%
Married	64.0	16.2%
Separated	21.0	5.3%
Single	191.0	48.4%
Widowed	8.0	2.0%
**Highest level of education**		
Completed grade 12	222.0	56.2%
No formal education	4.0	1.0%
Primary school	32.0	8.1%
Secondary	89.0	22.5%
Tertiary	48.0	12.2%
**Employment history**		
Full time employed	91.0	23.0%
Part-time employed	96.0	24.3%
Self-employed	26.0	6.6%
Unemployed	182.0	46.1%
**Residence**		
Hostel	13.0	3.3%
Informal settlement	75.0	19.0%
Township	307.0	77.7%

**Tuberculosis preventive therapy history and tuberculosis occurrence:** Table 2 shows that 30% (119/295) of the participants completed the TPT treatment. Tuberculosis was found among 3% (12/395) of the participants, half (6/12 (50%)) of which was TB that occurred before the initiation of TPT treatment, resulting in TB incidence of 6/389 (1.5%). The remaining six (6) TB cases occurred in the course of TPT treatment, with only 1 (16.7%) who had completed TPT. The results also indicate that participants took a median of 7 months on TPT (IQR= 8 months), with the minimum and the maximum time of 4 and 12 months, respectively. The median time between TPT initiation and TB diagnosis was 25 months (IQR=30 months), with a minimum and a maximum of 4 and 43 months, respectively.

**Table 2:** tuberculosis incidence and tuberculosis preventive therapy completion rate, N=395

Characteristic	Frequency	Percentage
**Tuberculosis preventive therapy completion at 12 months**		
Yes	119	30.1%
No	276	69.9%
**Tuberculosis occurrence**		
Yes	12	3.0%
No	383	97.0%
**Timing of tuberculosis occurrence, n=12**		
Before tuberculosis preventive therapy initiation	6	50%
In the course of tuberculosis preventive therapy	6	50%
**Tuberculosis incidence, n=389**	6	1.5%
**Tuberculosis preventive therapy completion at 12 months among participants who developed tuberculosis during the course of tuberculosis preventive therapy, n=6**		
Yes	1	16.7%
No	5	83.3%
**Time taken to complete tuberculosis preventive therapy (months)**		
Minimum	4	
Median (IQR)	7 (8)	
Maximum	12	
**The time period between tuberculosis preventive therapy initiation and tuberculosis diagnosis (months)**		
Minimum	4	
Median (IQR)	25 (30)	
Maximum	48	

**Reasons for non-tuberculosis preventive therapy completion:** analysis of the reasons for non-completion of TPT shows that out of the 276 participants who did not complete the treatment at the recommended 12 months, one hundred and seven (39%)) were not offered the treatment by the clinicians. Other reasons include unavailability of TPT at the health facility 46 (17%), work commitments 43 (15%), a health facility that is far 14 (5.0%), and the treatment side effects 11 (4%). The remaining 55 (20%) reasons include participants who were not comfortable with coming to the clinic, poor staff attitude, and being lazy, Table 3.

**Table 3:** reasons for non-completion of tuberculosis preventive therapy at 12 months, n=276

Reasons for tuberculosis preventive therapy non-completion	Frequency	Percentage
Clinician did not give	107	39%
Tuberculosis preventive therapy not available	46	17%
Work commitments	43	15%
Health facility far	14	5%
Treatment side-effects	11	4%
**Other, n=55**		
laziness	14	25%
Poor staff attitude	17	31%
Discomfort coming to the clinic	24	44%

**Barriers and facilitators to tuberculosis preventive therapy completion:** in Table 4 shows the responses to questions pertaining to barriers and facilitators of TPT completion. The results indicate that 99.5% (295/393) of the participants did not feel overburdened with the pills. Most did not have a treatment supporter (239 (60.5%)) while 264 (66.8%) had disclosed their positive HIV status. The majority reported experiencing side effects, (96.7% (382)). Two hundred and sixty-four (66.8%) indicated they had never received information on TPT. Most of the participants were comfortable taking TPT, (317 (80.3%)), with 74.2% (293) who thought the staff attitude was good. The majority lived at a distance ≤ 5 km from a health facility (345 (87.3%)), with (386 (97.7%)) who took ≤ 1 hour to travel to a health facility. Slightly more than half of the participants thought consultations at the health facility lasted > 3 hours (220 (55.7%)). TPT completion rates were higher among participants who: i) did not feel overburdened by the pills, (118 (99.2%)); ii) had a treatment supporter, (82 (68.9%)); disclosed the positive HIV status, (99 (83.2%)); iii) did not experience side effects, (115 (96.6%)); iv) ever received information about TPT, (80 (67.2%)); v) were comfortable taking TPT, (111 (93.3%)); vi) thought the staff attitude was good,(100 (84%)); vii) lived at a distance a distance ≤5 km from the health facility, (104 (87.4%)); viii) traveled ≤ 1 hour to a health facility, (117 (98.3%)); viv) and participants who thought consultation hours took 3 hours, (62 (52.1%)). Analysis of the barriers and facilitators showed that not having a treatment supporter and not ever receiving information about TPT were barriers to TPT completion, (202/276 (73.2%)), p<0.001) and 225/276 (81.5%)), p <0.001. Disclosure of a positive HIV status, being comfortable in taking TPT, and reporting good staff attitude were observed to facilitate TPT completion, respectively, 99/119 (83.2%); 111 (93.3%) and 100/119 (100 (84%), all statistically significant, p <0.001.

**Table 4:** barriers and facilitators to tuberculosis Preventive therapy completion, N=395

Question item	Total N=395	Tuberculosis preventive therapy completed		P-value
		**Yes, n=119**	**No, n=276**	
**Do you feel like you are overburdened with pills?**				
Yes	2 (0.5%)	1 (0.8%)	1 (0.4%)	0.51
No	393 (99.5%)	118 (99.2%)	275 (99.6%)	
**Do you have a treatment supporter?**				
Yes	156 (39.5%)	1 (0.8%)	74 (26.8%)	<0.001
No	239 (60.5%)	37 (31.1%)	202 (73.2%)	
**Have you disclosed your positive HIV status?**				
Yes	264 (66.8%)	99 (83.2%)	165 (59.8%)	<0.001
No	131 (33.2%)	20 (16.8%)	111 (40.2%)	
**Have you ever experienced side effects?**				
Yes	2 (0.5%)	1(0.8%)	1 (0.4%)	0.87
No	382 (96.7%)	115 (96.6%)	267 (96.7%)	
I don’t know	11 (2.8%)	3 (2.5%)	8 (2.9%)	
**Have you ever received information about tuberculosis preventive therapy?**				
Yes	131 (33.2%)	80 (67.2%)	51 (18.5%)	<0.01
No	264 (66.8%)	39 (32.8%)	225 (81.5%)	
**Are you comfortable taking tuberculosis preventive therapy?**				
Yes	317 (80.3%)	111 (93.3%)	206 (74.6%)	<0.001
No	17 (4.3%)	5 (4.2%)	12 (4.3%)	
I don’t know	61 (15.4%)	3 (2.5)	58 (21.0%)	
**How is the facility staff attitude?**				
Good	293 (74.2%)	100 (84%)	193 (69.9%)	<0.01
Bad	19 (4.8%)	3 (2.5%)	16 (5.8%)	
Depends on the clinician	83 (21%)	16 (13.4%)	67 (24.3%)	
**How far is your home from the health facility (in km)?**				
≤5 km	345 (87.3%)	104 (87.4%)	241 (87.3%)	1.00
>5 km	50 (12.7%)	15 (12.6%)	35 (12.7%)	
**How long does it take you to travel to the health facility (in minutes)?**				
≤1 hour	386 (97.7%)	117 (98.3%)	269 (97.5%)	0.72
>1 hour	9 (2.3%)	2 (1.7%)	7 (2.5%)	
**Average consultation**				
≤hours	175 (44.3%)	57 (47.9%)	118 (42.8%)	0.37
>3 hours	220 (55.7%)	62 (52.1%)	158 (57.2%)	

## Discussion

The 60% female majority observed in this study corroborates findings from other studies which reported females constituting between 58.5% and 70% of the participants [17,16,19-21]. Others have similarly reported lower ART access among males compared to females [22]. The mean age of the participants in this study was 39 years. Similarly, others have reported mean ages ranging between 32 years and 41 years [16,17,23]. This study found nearly half of the unemployed participants (46%). Much higher unemployment rates of 60% and above have been reported among study participants [17]. The study determined the proportion of patients who completed TPT at the prescribed 12 months and found 30%. Similarly, others have reported completion rates of 30% and lower [8,16], however, others have reported completion rates as high as 92.8% among PLWHA [24]. This study found that 39% of participants did not complete TPT because it had not been offered TPT by the clinicians. Others have reported skepticism among healthcare workers (HCWs) about TPT as a medical intervention and competing deprioritization of TPT to prioritize other patient concerns [4]. The finding of availability of the TPT treatment. Similarly, others have reported TPT stockouts as barriers to TPT non-completion [25,26]

This study assessed the TB incidence among the 395 participants and found that 3% had a positive TB diagnosis, of which 50% had TB that occurred during TPT treatment. Five out of the six breakthrough cases of TB were probably due to suboptimal treatment as these were participants who did not complete the prescribed 12 months for the TPT treatment course. Other studies have reported proportions of breakthrough TB as low as 0.3% and 0.5% [27,28]. The study further investigated the barriers and facilitators of TPT completion. The results showed that not having a treatment supporter was a barrier to TPT completion, whilst disclosure of a positive HIV status, feeling comfortable in taking TPT, and having a good staff attitude were all found to facilitate TPT completion. Several studies have reported improved treatment adherence among patients who had treatment support, and those who had disclosed positive HIV status [29-31]. The findings of this study are worrisome as PLWHA are at high risk of developing TB, and those who are on TPT but not completing it are at risk of breakthrough TB. This can have some implications on public health due to a high number of emerging TB cases and can place a burden on the healthcare system.

**Limitations:** this study had limitations which included a small sample size and incomplete data. The study also excluded patients who were using external pick-up points, which could have resulted in an underestimation of the TPT completion rate.

## Conclusion

The low TPT completion rate observed in this study is far below the recommended national threshold of 85%. Failure to address this shortfall will lead to an increase in the TB/HIV coinfection rate in South Africa, a country that is overburdened with the prevalence of TB and HIV. The finding of clinicians who do not offer TPT to qualifying HIV-positive patients is worrisome as this contributes to suboptimal uptake of TPT; this needs to be explored with further research. TPT stockouts need to be addressed at a health facility level to avoid delayed initiation of individuals who qualify for this intervention, and consequent incidence of TB among those who might have been initiated on TPT already. The finding of participants who were initiated on TPT but had never received information about TPT requires urgent attention as this lack of patient literacy concerning TPT may contribute to the non-completion of this intervention.

### What is known about this topic

- *tuberculosis preventive therapy implementation is one of the major hurdles in TPT uptake;*
- *tuberculosis preventive therapy completion is essential for the prevention of TB incidence among PLWHA who do not show the signs and symptoms of active TB disease, yet*.

### What this study adds

- *Some participants in this study did not complete TPT because it was not offered by the clinicians;*
- *The majority of participants who developed breakthrough TB in this study had not completed the TPT course of treatment*.
